# Endosymbiotic *Chlorella variabilis* reduces mitochondrial number in the ciliate *Paramecium bursaria*

**DOI:** 10.1038/s41598-022-12496-8

**Published:** 2022-05-30

**Authors:** Yuuki Kodama, Masahiro Fujishima

**Affiliations:** 1 Institute of Agricultural and Life Sciences, Academic Assembly, Shimane University, 1060 Nishikawatsu-cho, Matsue-shi, Shimane 690-8504 Japan; 2 Yamaguchi University, Yoshida 1677-1, Yamaguchi, 753-8511 Japan

**Keywords:** Microbiology, Cellular microbiology, Microbial communities, Environmental microbiology

## Abstract

Extant symbioses illustrate endosymbiosis is a driving force for evolution and diversification. In the ciliate *Paramecium bursaria*, the endosymbiotic alga *Chlorella variabilis* in perialgal vacuole localizes beneath the host cell cortex by adhesion between the perialgal vacuole membrane and host mitochondria. We investigated whether host mitochondria are also affected by algal endosymbiosis. Transmission electron microscopy of host cells showed fewer mitochondria beneath the algae-bearing host cell cortex than that of alga-free cells. To compare the density and distribution of host mitochondria with or without symbiotic algae, we developed a monoclonal antibody against *Paramecium* mitochondria. Immunofluorescence microscopy with the monoclonal antibody showed that the mitochondrial density of the algae-bearing *P. bursaria* was significantly lower than that of the alga-free cells. The total cell protein concentration of alga-free *P. bursaria* cells was approximately 1.8-fold higher than that of algae-bearing cells, and the protein content of mitochondria was significantly higher in alga-free cells than that in the algae-bearing cells. These results corresponded with those obtained by transmission electron and immunofluorescence microscopies. This paper shows that endosymbiotic algae affect reduced mitochondrial number in the host *P. bursaria* significantly.

## Introduction

Mitochondria and the photosynthetic plastids in eukaryotic cell were derived from once free-living prokaryotic cells, which symbiotically acquired by some early cells^1^. This endosymbiosis process provided plants for animals to eat and additional oxygen for their breathe and thus Earth has been changed drastically^2^. Endosymbionts in protists help host cells with new biochemical processes, such as photosynthesis, nitrogen fixation, nitrogen recycling, methanogenesis, and sulfide oxidation^3^. The ciliate *Paramecium bursaria* harbors approximately 700 symbiotic *Chlorella* spp. cells in its cytoplasm, and the ciliate is one example which shows symbioses as a mechanism of evolutionary innovation^4^. These algae are enclosed in a symbiosome called the perialgal vacuole (PV) membrane, derived from the host digestive vacuole (DV) membrane. The PV membrane protects the algae from host lysosomal fusion^5–7^. Symbiotic algae supply the host with photosynthetic products, mainly maltose^8–11^, while the host provides the algae with nitrogenous compounds and CO_2_^9,12–14^. Uniquely, irrespective of the mutual relationship between *P. bursaria* and *Chlorella* spp., their relationship is not obligatory. Each can grow independently under suitable conditions. In addition, alga-free *P. bursaria* can be reinfected with isolated symbiotic algae by ingestion of the algae into host DVs. Based on these characteristics, *P. bursaria* and *Chlorella* spp. are considered textbook examples of endosymbiosis in protists^3^.

In previous studies, by pulse-labeling alga-free *P. bursaria* for 1.5 min with symbiotic algae isolated from algae-bearing paramecia and then chasing at specific times, we identified four important cytological events necessary for the establishment of endosymbiosis, as well as the timings of each during the algal infection process^7,15–23^. These four cytological events are described below. (i) Three minutes after algal mixing, part of the algae is resistant to the host’s lysosomal digestive enzymes in the DVs^16,18^. (ii) Within 30 min of mixing, algae in the DV begin budding from the DV membrane and enter the cytoplasm^16,20^. (iii) Fifteen minutes after budding, the DV membrane enclosing a single green *Chlorella* differentiates into a PV membrane, protecting the alga from host lysosomal fusion^16,18^. (iv) The alga surrounded by a PV membrane translocates beneath the host cell cortex^16,22,24^. Furthermore, the PV appears to localize near the host mitochondria and trichocysts^25^. Trichocysts are defensive organelles against predators embedded in the *Paramecium* cell cortex^26^. Indirect immunofluorescence microscopy using a monoclonal antibody (mAb) against the trichocysts demonstrates that the trichocysts change their localization to form algal attachment sites and decrease their number beneath the host cell cortex through algal reinfection^22^. Transmission electron microscopy (TEM) showed that some trichocysts near the host cell cortex were digested by host lysosomal fusion during algal reinfection^22^. These results indicate that symbiotic algae compete for their attachment sites with the pre-existing trichocysts and ensure attachment beneath the host cell cortex^22^.

Many mitochondria are observed around the symbiotic *Chlorella* sp., similar to trichocysts in algae-bearing *P. bursaria*, and the symbiotic algae adhere to host mitochondria^25^. About host mitochondria in symbiotic state, Reisser reported that symbiotic algae in *P. bursaria* show a higher rate of photosynthetic oxygen production than those in the isolated state and thus guarantee an oxygen supply for the host^14^. Furthermore, recently He et al. also showed that the endosymbiotic algae may produce sufficient oxygen through their photosynthesis for the host cells to maintain cellular respiration in mitochondria^27^. Therefore, we had considered that the presence of symbiotic algae wrapped in the PV membrane may affect the number and function of host mitochondria. Song et al. reported that the PV membrane directly contacts the *P. bursaria*’s mitochondrial membrane^28^. Similar results were observed in *Mayorella visiris*^29,30^, *Toxoplasma gondii*^31^, *Plasmodium* sporozoites^32^, and *Encephalitozoon* microsporidia^33^. Therefore, the association of host mitochondria with symbionts may be a universal component in symbiotic relationships.

Here, we observed cell cortex of alga-free and -bearing *P. bursaria* cells using TEM to examine the relationships between host mitochondria and symbiotic algae wrapped with the PV membrane. To examine whether the host mitochondria were affected by the endosymbiotic algae, we developed a mAb against *Paramecium* mitochondria. The relationships between the distribution of the symbiotic algae and the density of mitochondria were analyzed by an indirect immunofluorescence microscopy using mAb. The immunofluorescence localization was compared using the fluorescence of a mitochondria-specific dye, MitoTracker Green FM. Furthermore, the total cell protein concentration and that of the four kinds of cell extracts, including the mitochondrial membrane, was examined in the presence or absence of symbiotic algae. From the previous transcriptome data of host *P. bursaria*^34^, the expression levels of mitochondria-related genes were compared in the presence or absence of symbiotic *Chlorella variabilis*.

## Methods

### Strains and cultures

The symbiotic *Chlorella* sp.-free (alga-free) *P. bursaria* strain Yad1w was produced from the *Chlorella* sp.-bearing (algae-bearing) *P. bursaria* strain Yad1g as described previously^18^. The algae-bearing Yad1g1N strain was produced by infecting Yad1w cells with cloned symbiotic *Chlorella* sp. 1 N cells^22^. This strain was identified as *C. variabilis* by rbcL gene analysis (accession number PRJDB12213, https://ddbj.nig.ac.jp/resource/bioproject/PRJDB12213 ). Both alga-free and algae-bearing *P. bursaria* cells were cultured in red pea (*Pisum sativum*) extract culture medium^35^ with a modified Dryl’s solution^36^ (KH_2_PO_4_ was used instead of NaH_2_PO_4_·2H_2_O) and inoculated with *Klebsiella aerogenes* (strain ATCC 35028) 1 day before use. In all cultures, several hundred *P. bursaria* cells were inoculated into 2 mL aliquots of culture medium in test tubes. Subsequently, 2 mL aliquots of fresh culture medium were added every day and were cultivated for 12 days. The next day, the cultures reached to the early stationary phase of growth. All the cells used in this study were at this phase. Cultivation of the algae-bearing *P. bursaria* strain and all experiments were performed at 23 °C ± 1 °C under fluorescent lighting at 20–30 μmol photons m^-2^ s^-1^ using an incandescent lamp. Alga-free *P. bursaria* were cultivated under the same conditions, without lighting. All *Paramecium* strains used in this study were provided by the NBRP *Paramecium* Laboratory, Yamaguchi University, with support, in part, by the NBRP of the Ministry of Education, Culture, Sports, Science, and Technology (MEXT) (http://nbrpcms.nig.ac.jp/paramecium/?lang=en).

### TEM

Both algae-bearing and alga-free *P. bursaria* were pre-fixed with 2% glutaraldehyde and prepared for TEM as described previously^37^. The paramecia embedded in Spurr’s resin^38^ were sectioned (70 nm thickness) using an ultramicrotome (Reichert Ultracut S; Leica Microsystems, Vienna, Austria) with a diamond knife, mounted on nickel mesh grids, and stained with lead citrate^39^. The sections were observed using TEM (CM120; Philips) at 80 kV.

### Production of a mAb specific for *Paramecium* mitochondria

We produced a mitochondria-specific mAb as described in our previous research article^22^. The homogenate of algae-bearing *P. bursaria* was injected into the peritoneal cavity of an 8-week-old BALB/c mouse. A hybridoma clone, mAb2B8A8H1, was used in this study. Hybridoma cell production was performed following the guidelines for the use of animals in research at Yamaguchi University.

### Indirect immunofluorescence microscopy

Indirect immunofluorescence microscopy was performed as described previously^22^. Aliquots of *P. bursaria* cells were air-dried on cover glasses (4.5 × 24 mm). The cells were then fixed with 4% (w/v) paraformaldehyde in phosphate-buffered saline (PBS) (137 mM NaCl, 2.68 mM KCl, 8.1 mM NaHPO_4_·12H_2_O, 1.47 mM KH_2_PO_4_, pH 7.2) for 10 min at 4 °C. Then, the fixed cells were washed with PBST (PBS containing 0.05% Tween 20) and PBS for 10 min at 4 °C. The cells were then treated with mAb overnight at 4 °C. The cells were washed twice with PBS. Next, the cells were treated with Alexa Fluor 488 (AF488) goat anti-mouse IgG (Molecular Probes) diluted 1,000-fold with PBS for 2 h at 23 °C ± 1 °C and washed twice with PBS for 10 min. The samples were observed under differential interference contrast (DIC) and fluorescence microscopy (BX53; Olympus, Tokyo, Japan) equipped with Olympus fluorescence mirror units U-FBNA (excitation 470–495 nm, emission 510–550 nm) for AF488 and U-FGW (excitation 530–550 nm, emission 575 nm) for algal autofluorescence. Images were acquired using an Olympus DP74 system and analyzed using the Olympus cellSens Dimension software.

### Visualization of *P. bursaria* mitochondria with MitoTracker Green

MitoTracker Green was obtained from Molecular Probes (Eugene, OR, USA) and stored at –20 °C until further use. To stain the mitochondria with this reagent, approximately 5,000 algae-bearing *P. bursaria* cells/ml in MDS were mixed with MitoTracker Green at a concentration of 500 nM under constant dark conditions at 23 °C ± 1 °C, and the cells were observed using DIC and fluorescence microscopy within 10 min after mixing.

### Protein quantification of the host *P. bursaria* with or without symbiotic algae

Alga-free and algae-bearing *P. bursaria* cells in the early stationary phase of growth were strained through two layers of Kimwipes to remove gross debris. The cells were transferred to a plastic beaker equipped with nylon mesh with a pore size of 15 μm. The paramecia were harvested, and the cell pellet was washed by pouring 100 mL PBS into the plastic beaker. The total cell extract was obtained as follows. Alga-free or algae-bearing *P. bursaria* at a density of 5 × 10^4^ cells were lysed using the EzRIPA Lysis kit (ATTO, Tokyo, Japan) according to the manufacturer’s instructions. After centrifugation, the supernatant was stored at − 80 °C until analysis. Extracts of the cytoplasm, including mitochondrial membrane, nucleus, and insoluble part (i.e., cytoskeleton) of *P. bursaria* cells were obtained as follows. Washed and harvested alga-free or algae-bearing *P. bursaria* at a density of 5 × 10^4^ cells were treated with EzSubcell Extract (ATTO), and each extract was prepared according to the manufacturer’s instructions. The extracts were stored at − 80 °C until analysis. Protein concentrations of total cell lysates and four types of cell extracts (cytoplasm, mitochondrial, nucleus, and insoluble part) were determined using the TaKaRa BCA Protein Assay Kit (Takara Bio, Shiga, Japan). Calibration curves were generated using PiCOEXPLORER (Yamato Scientific Co., Ltd., Japan, PAS-110-YU) with a color sensor R (wavelength range, 575–660 nm).

### Quantification and statistical analysis

Statistical analyses were performed using Microsoft® Excel for Mac (Ver. 16.51). Quantitative data were analyzed using the two-sided Fisher’s exact test. For immunofluorescence intensity (a.u.), the statistical significance was determined using two-sided Fisher’s exact test and the data of 10–12 cells. This experiment was repeated more than 10 times, and similar statistical results were confirmed. For the quantification of protein concentration, statistical significance was determined using two-sided Fisher’s exact test and the data from four to five independent experiments.

## Results

### TEM of alga-free and -bearing *P. bursaria*

**Figure 1:**
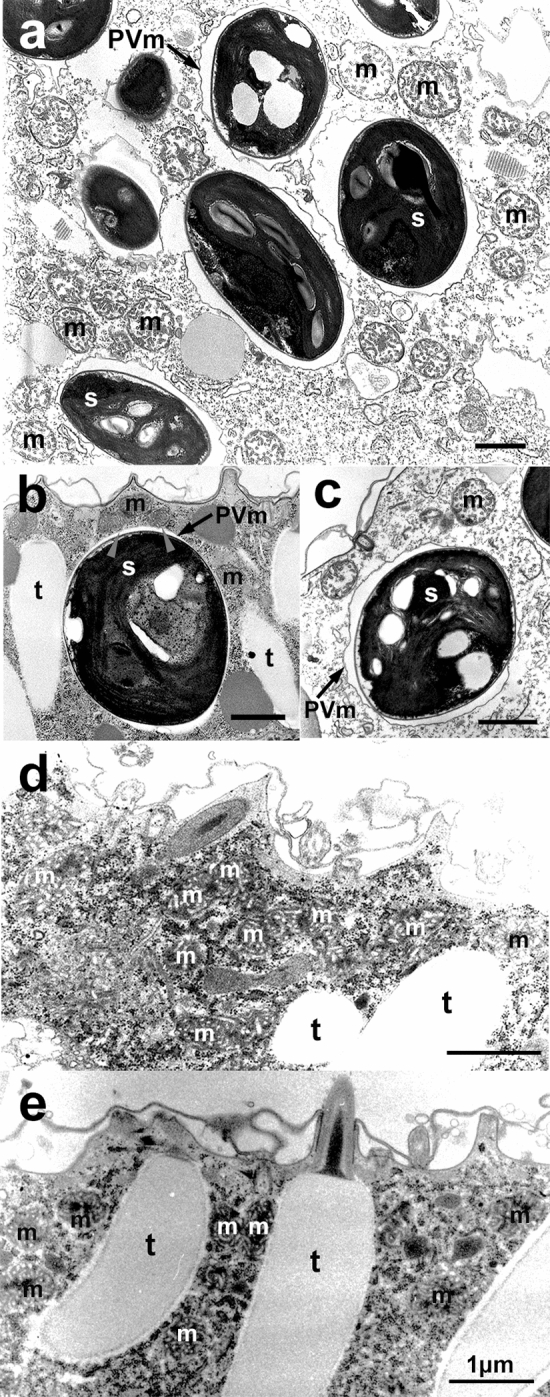
Transmission electron microscopic images of the cell cortex of algae-bearing (**a**)–(**c**) and alga-free (**d**) and (**e**) *P. bursaria* cells. (**a**) Host mitochondria surround the PV membrane wrapping the symbiotic alga (s). (**b**) Symbiotic alga between the trichocysts beneath the host cell cortex. (**c**) Both host trichocysts and mitochondria are very few around the symbiotic alga. Gray arrowheads in (**b**) indicate mitochondria attached to the PV membrane. Note that many mitochondria and trichocysts can be observed beneath the cell cortex in alga-free cells (**d**) and (**e**). t, trichocyst; m, mitochondrion; PVm, PV membrane; s, symbiotic alga. Bars = 1 μm.

The cell cortex of the algae-bearing and alga-free *P. bursaria* were observed using TEM (Fig. 1). The host mitochondria surrounded the PV membrane wrapping the symbiotic alga (Fig. 1a). The symbiotic alga appeared to push the trichocysts aside to become fixed near the host cell cortex, as shown in our study^7^ (Fig. 1b). In some algae-bearing cells, both host trichocysts and mitochondria were very few around the symbiotic alga beneath the host cell cortex (Fig. 1c). As shown by the gray arrowheads in Fig. 1b, we could observe mitochondria attached to the PV membrane, as observed in previous studies^25,28^. Meanwhile, in alga-free cells, many mitochondria and trichocysts were observed beneath the cell cortex (Fig. 1d). Mitochondria filled the gap just below the host cell cortex (Fig. 1e).

### mAb specific for *P. bursaria* mitochondria

**Figure 2:**
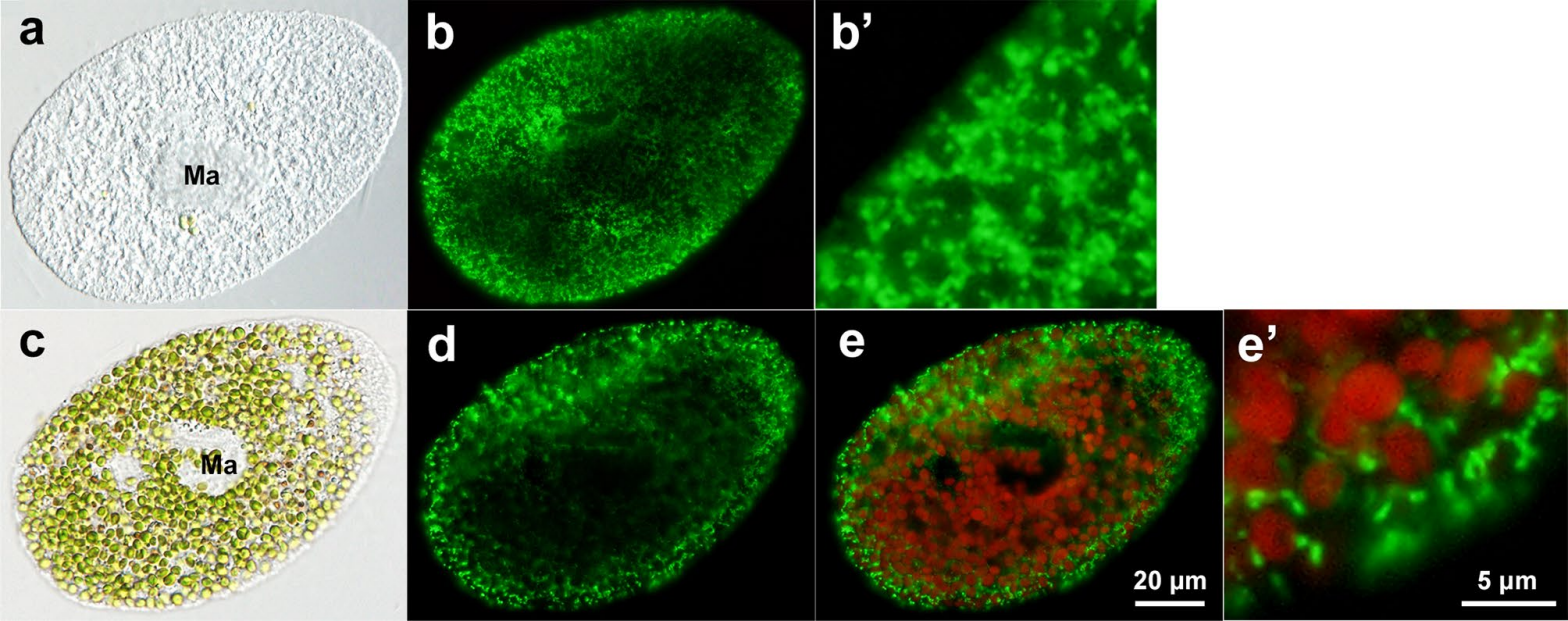
Indirect immunofluorescence micrographs of alga-free *P. bursaria* strain Yad1w cell (**a**)–(**b’**) and algae-bearing *P. bursaria* strain Yad1g1N cell in (**c**)–(**e’**). (**a**) and (**c**): DIC micrographs. (**b**) and (**d**): Images of immunofluorescence of goat anti-mouse IgG. (**b’**) Enlargement the host cell cortex in (**b**). (**e**) A merged image of (**d**) and the autofluorescence of chlorophyll in algal chloroplasts. (**e’**) The enlargement of some symbiotic algae near the host cell cortex in (**e**). Note that the host mitochondria are localized around the symbiotic algae (**e’**). Immunofluorescence shows that the mitochondria of the cell cortex of the alga-free cells are more numerous (**b’**) than those of algae-bearing cells (**e’**). Ma, macronucleus. Bar = 20 μm (**a**, **b**, **c**, **d**, and **e**) and 5 μm (**b’**and **e’**).

We successfully obtained a mAb specific for *Paramecium* mitochondria. Immunofluorescence was observed in the entire cell, particularly in the paramecium cell cortex (Fig. 2b,d). In the case of algae-bearing cells, fluorescence was not observed in areas where symbiotic algae were located (Fig. 2d). Immunofluorescence showed a higher number of mitochondria in the alga-free *P. bursaria* cells (Fig. 2a,b’) than in the algae-bearing cells (Fig. 2c,e’). A merged photomicrograph of red autofluorescence of chlorophyll within the chloroplasts of the symbiotic algae and immunofluorescence clearly showed the localization of host mitochondria around symbiotic algae (Fig. 2e,e’). These immunofluorescence observations are in good agreement with the observations using TEM, as shown in Fig. 1. Images were obtained from a representative of more than ten independent experiments.

**Figure 3:**
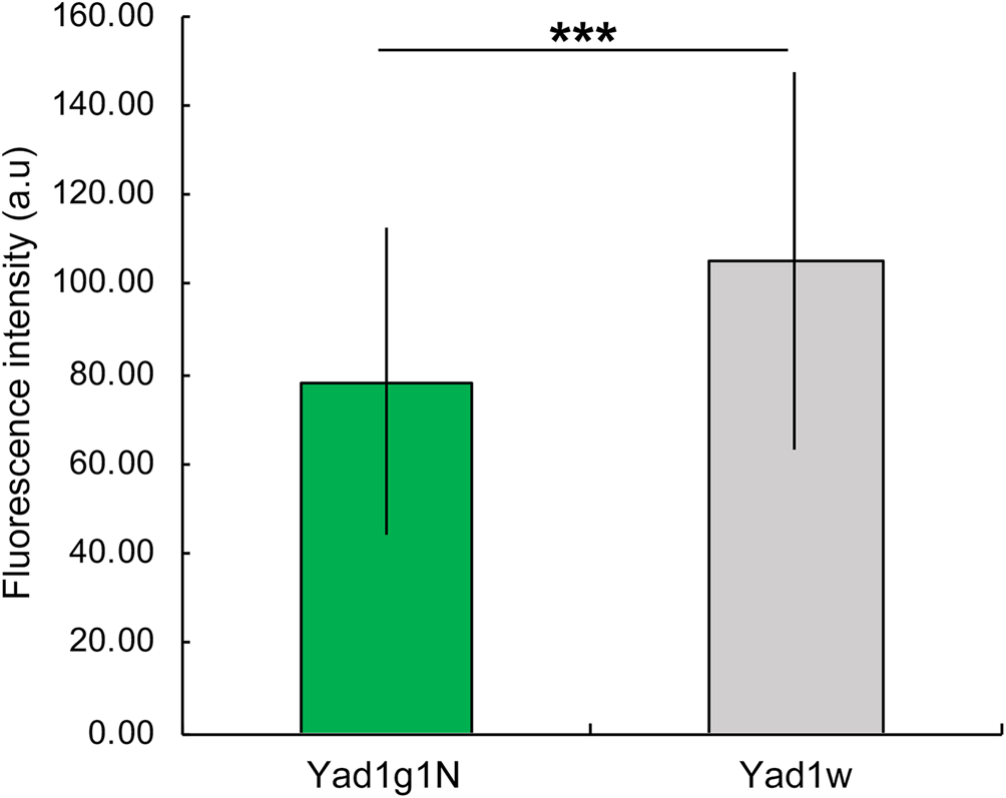
Immunofluorescence intensity of mitochondria of alga-free and algae-bearing *P. bursaria* cells. In alga-free *P. bursaria* cells (gray bar graph), the immunofluorescence intensity is greater than that of algae-bearing *P. bursaria* cells (green bar graph). Ten to twelve *Paramecium* cells were observed. Error bars show standard deviation (SD). Asterisks indicate significant differences (two-sided Fisher’s exact test, ***P < 0.001).

The immunofluorescence intensity of the alga-free *P. bursaria* cells (Fig. 3, gray bar graph), was greater than that of the algae-bearing cells (Fig. 3, green bar graph). These quantitative data correspond well with the results of indirect immunofluorescence microscopy, as shown in Fig. 2.

### Visualization of mitochondria in *P. bursaria* with MitoTracker Green

**Figure 4:**
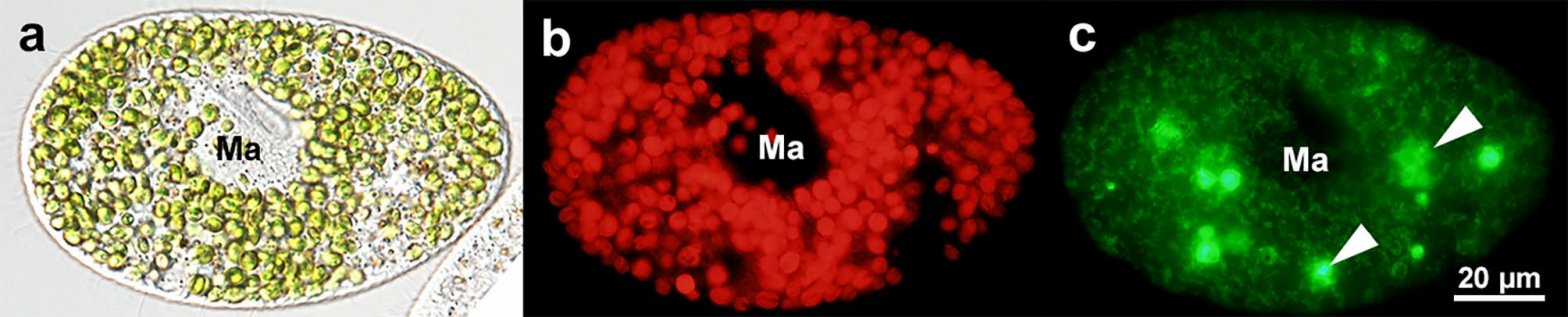
Photomicrographs of algae-bearing *P. bursaria* strain Yad1g1N cell with MitoTracker Green. (**a**) DIC micrograph. (**b**) Autofluorescence micrograph of chlorophyll in algal chloroplasts. (**c**) Fluorescence of MitoTracker Green. Host mitochondria appear to be surrounded symbiotic algae. Note that the fluorescence of MitoTracker Green is also observed in the host DVs (**c**, arrowhead). Ma, macronucleus. Bar = 20 μm.

MitoTracker Green FM passively diffuses across the cell membrane, accumulates in active mitochondria, and shows green fluorescence. Figure 4a, b show a DIC image and a red autofluorescence of chlorophyll within chloroplasts of symbiotic algae image, respectively. The host mitochondria were localized around symbiotic algae (Fig. 4c). The fluorescence pattern of mitochondria of MitoTracker Green was consistent with that obtained using the mAb against mitochondria. After staining with MitoTracker Green, some DVs were formed, and nonspecific fluorescence was observed in the DVs because *Paramecium* takes in anything other than food from its cytopharynx (Fig. 4c, white arrowheads). This nonspecific fluorescence affects the fluorescence quantification, but this non-specific fluorescence was not observed using the mAbs, indicating the utility of mAbs for mitochondrial visualization.

### Protein quantification of the host *P. bursaria* with or without symbiotic algae

**Figure 5:**
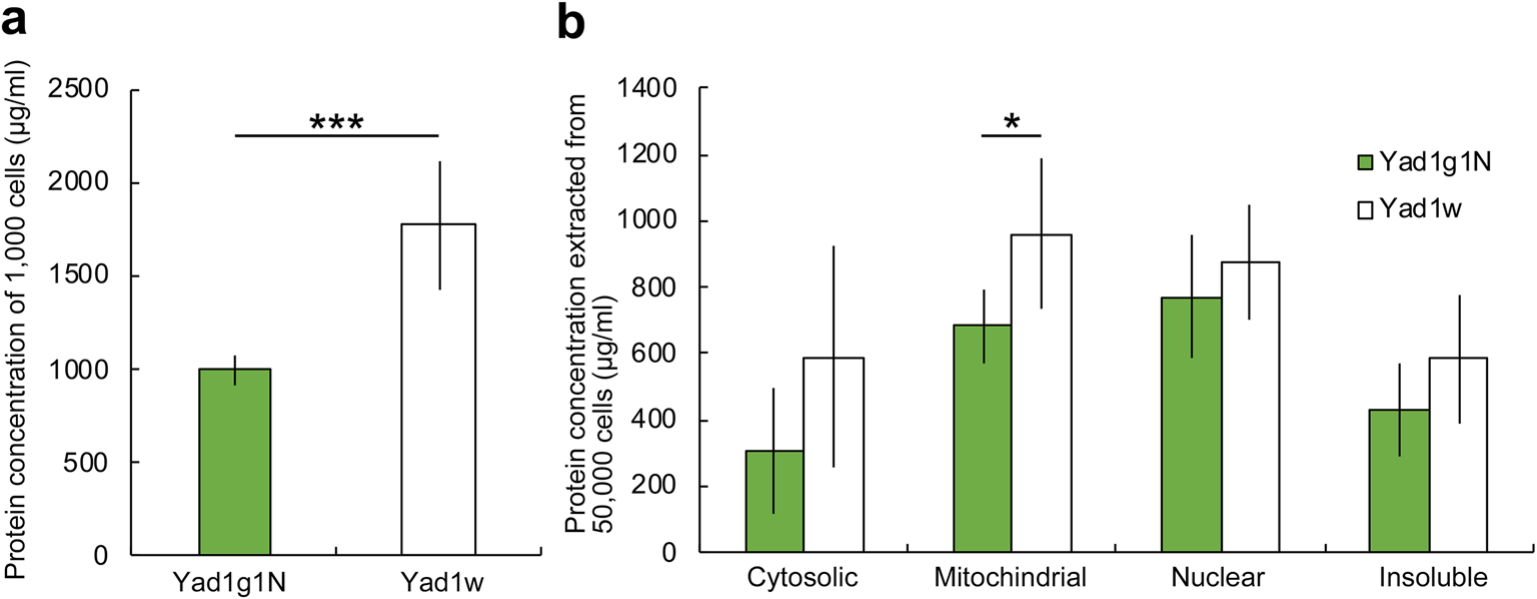
(**a**) Total cell protein concentrations of alga-free (white bar graph) and algae-bearing (green bar graph) *P. bursaria* cells. The protein concentration of alga-free *P. bursaria* cells was about 1.8 times higher than that of algae-bearing cells. (**b**) Protein concentration of each cell extract of alga-free (white bar graph) and algae-bearing (green bar graph) *P. bursaria* cells. The four extracts are cytosolic, including mitochondrial membrane, nuclear, and insoluble. The protein concentration including mitochondrial membrane of alga-free *P. bursaria* cells was significantly higher than that of algae-bearing cells. Error bars show the standard deviation (SD) obtained from experiments 5 (**a**) and 4–6 (**b**). Asterisks indicate significant differences (two-sided Fisher’s exact test, ***P <0.001, *P < 0.05).

First, the total cell protein concentrations of alga-free and algae-bearing *P. bursaria* were measured. Since the EzRIPA Lysis kit is used for animal cells, it cannot dissolve *Chlorella* cells. In fact, we confirmed that the isolated symbiotic *Chlorella* cells could not be dissolved using the EzRIPA Lysis buffer, and no protein was extracted (data not shown). The insoluble precipitates of alga-free and algae-bearing *P. bursaria* cells contained many intracellular crystals and symbiotic algae, respectively. We found that the protein concentration of 1,000 cells of alga-free *P. bursaria* cells was approximately 1.8-fold higher than that of algae-bearing cells (Fig. 5a). To understand the reason behind this significant difference in the concentrations, the protein concentrations of four extracts (cytosolic, including mitochondrial membrane, nuclear, and insoluble) from the 50,000 cells were examined. Of the four extracts, only the concentration of proteins of the mitochondrial extract was significantly different between alga-free and algae-bearing *P. bursaria* cells. The protein concentration including mitochondrial membrane of alga-free *P. bursaria* cells was significantly higher than that of algae-bearing cells (Fig. 5b). In the other three extracts, the protein concentration of alga-free *P. bursaria* cells was higher than that of algae-bearing cells; however, the difference was not significant. The protein concentration of the mitochondrial extract was in good agreement with the TEM and indirect immunofluorescence microscopy observations, as shown in Figs. 1, 2 and 3.

### Differential expression of mitochondria-related genes between algae-bearing and alga-free *P. bursaria*

Table 1 shows the transcripts encoding mitochondria-related proteins in *P. bursaria* from Kodama et al^34^. The negative values of log_2_fold change (log_2_FC) showed that mitochondrial genes were downregulated in algae-bearing cells compared to alga-free cells. The data sets are available in the DDBJ Sequence Read Archive (DRA) (accession number DRA000907, https://ddbj.nig.ac.jp/resource/sra-submission/DRA000907). These results were consistent with the results obtained by TEM, indirect immunofluorescence microscopy, and quantification of the mitochondrial protein.

**Table 1:** Transcripts encoding mitochondria related protein in *P. bursaria* (from Kodama et al. ^34^).

Trinity transcript name	Annotation from the SwissProt database	logFC
comp37147_c1	sp|P05489|COX1_PARTE|Cytochrome c oxidase subunit 1 OS = *Paramecium tetraurelia*	− 1.146
comp40817_c0	sp|Q54MJ7|ALAM_DICDI|Probable alanine aminotransferase, mitochondrial OS = *Dictyostelium discoideum*	− 3.594
comp38395_c0	sp|Q8LCU7|MECR_ARATH|Probable trans-2-enoyl-CoA reductase, mitochondrial OS = *Arabidopsis thaliana*	− 2.757
comp38551_c0	sp|Q54KB7|DHE3_DICDI|Glutamate dehydrogenase, mitochondrial OS = *Dictyostelium discoideum*	− 4.319
comp44906_c0	sp|Q99MR8|MCCA_MOUSE|Methylcrotonoyl-CoA carboxylase subunit alpha, mitochondrial OS = *Mus musculus*	− 2.286
comp21987_c0	sp|Q3ZCF5|OAT_BOVIN|Ornithine aminotransferase, mitochondrial OS = *Bos taurus*	− 3.272
comp45458_c0	sp|P53395|ODB2_MOUSE|Lipoamide acyltransferase component of branched-chain alpha-keto acid dehydrogenase complex, mitochondrial OS = *Mus musculus*	− 2.053
comp37156_c0	sp|P40513|MAM33_YEAST|Mitochondrial acidic protein MAM33 OS = *Saccharomyces cerevisiae* (strain ATCC 204,508 / S288c)	− 2.015
comp37891_c0	sp|Q8BH95|ECHM_MOUSE|Enoyl-CoA hydratase, mitochondrial OS = *Mus musculus*	− 1.554
comp39058_c0	sp|Q9DCS3|MECR_MOUSE|Trans-2-enoyl-CoA reductase, mitochondrial OS = *Mus musculus*	− 1.775
comp45207_c0	sp|Q9FS87|IVD2_SOLTU|Isovaleryl-CoA dehydrogenase 2, mitochondrial (Fragment) OS = *Solanum tuberosum*	− 1.503
comp40488_c0	sp|Q43298|CH62_MAIZE|Chaperonin CPN60-2, mitochondrial OS = *Zea mays*	− 3.360

## Discussion

In our study, TEM and indirect immunofluorescence microscopy using a mAb against *Paramecium* mitochondria revealed that host mitochondria are located in the proximity of symbiotic algae (Figs. 1 and 2). As shown in “Introduction” Section, because the symbiosome membrane seems to contacts the host’s mitochondrial membrane directly^28–33^, the localization of the endosymbionts can be regarded as the same as that of the host mitochondria. The localization of the endosymbionts near the host cell cortex is a universal phenomenon because it is also observed in other ciliate–algae or ciliate–cyanobacteria endosymbiosis, e.g., *Mayorella viridis*, *Coleps hirtus*, *Coleps spetai*, *Frontonia leucas*, *Malacophrys sphagni*, *Ophrydium versatile*, *Vorticella* sp., *Climacostomum virens*, *Euplotes daidaleos*, *Halteria bifurcata*, *Stentor polymorphus*, and *Stentor niger*^10^. In general, most symbiotic algae do not flow in the cytoplasmic stream of *P. bursaria* but instead attach beneath the cell cortex. It is known that symbiotic algae fail to localize beneath the host cell cortex of mutant *P. bursaria* and form clusters in the host cytoplasm. This mutant cell passes an unequal distribution of the algal clusters to the daughter cell^40^. This suggests that the algal localization beneath the host cell cortex guarantees an equal distribution of symbiotic algae to daughter cells. Algal localization is an important phenomenon because the synchronization of host–symbiont cell cycles and co-segregation is critical in the permanent fusion of the two partners^41^. *Hatena arenicola* (Katablepharidophycota) is a single-celled eukaryote that harbors *Nephroselmis*. Because *H. arenicola* is regarded as an intermediate step organism in plastid acquisition, only one daughter cell inherits the symbiont during cell division, resulting in a symbiont-bearing green cell and a symbiont-lacking colorless cell. Interestingly, the symbiont-lacking cells have a feeding apparatus corresponding to the location of the eyespot in symbiont-bearing cells, and they can feed on prey cells^41–43^. In *P. bursaria–Chlorella* endosymbiosis, the symbiotic algae fine-tune their cell cycle pace with host *Paramecium*^44^. The symbiotic algal attachment in *P. bursaria* may be related to avoiding host lysosomal digestion or fusion because when the symbiotic algae are attached beneath the host cell cortex, the digestion never occurs (Kodama and Fujishima, unpublished. data).

In some cases, the presence of symbiotic algae diminishes the mitochondrial activity in the host. Among symbiotic algae-bearing *Hydra viridissima* downregulated genes, mitochondria-related genes were found to be enriched using the Gene Ontology (GO) terms during differential gene expression analysis. Furthermore, most of the genes involved in the respiratory chain were significantly downregulated in symbiotic algae-bearing *H. viridissima*^45^. Transient suppression of mitochondrial metabolism and protein synthesis occurred when *Acropora digitifera* planulae were exposed to a competent strain of symbiotic algae, *Symbiodinium*^46^. The degradation of host mitochondria was verified when the symbiotic sea anemone *Aiptasia pulchella* was cultivated under hyperthermic stress conditions independent of symbiont cellular deterioration^47^. Reactive oxygen species (ROS) generated by symbionts and mitochondria have been shown to play a central role in damage to the host, such as in coral bleaching^47^. Therefore, hosts with symbiotic algae are believed to have reduced mitochondrial activity to suppress ROS production.

In a previous study, we showed that glutathione S-transferase (GST)-encoding genes were downregulated in symbiont-bearing cells compared to symbiont-free cells using transcriptome analysis^34^. This enzyme protects cells from oxidative stress^48,49^. Although it is conceivable that photo-oxidative stress in algae-bearing *P. bursaria* cells is greater than that in alga-free cells^50^, our previous data showed opposite results. Although a similar result of us was obtained by Hörtnagl and Sommaruga^51^, the exact mechanism remains unknown. This is the first study to report that algae-bearing *P. bursaria* cells have fewer mitochondria than alga-free cells do. Since algae-bearing *P. bursaria* receives photosynthetic products from symbiotic algae, a large number of mitochondria is not required. Because algae-bearing *P. bursaria* has fewer mitochondria than those of alga-free cells, the amount of ROS in algae-bearing cells may also be low. This might explain the suppression of GST in algae-bearing *P. bursaria*.

**Figure 6:**
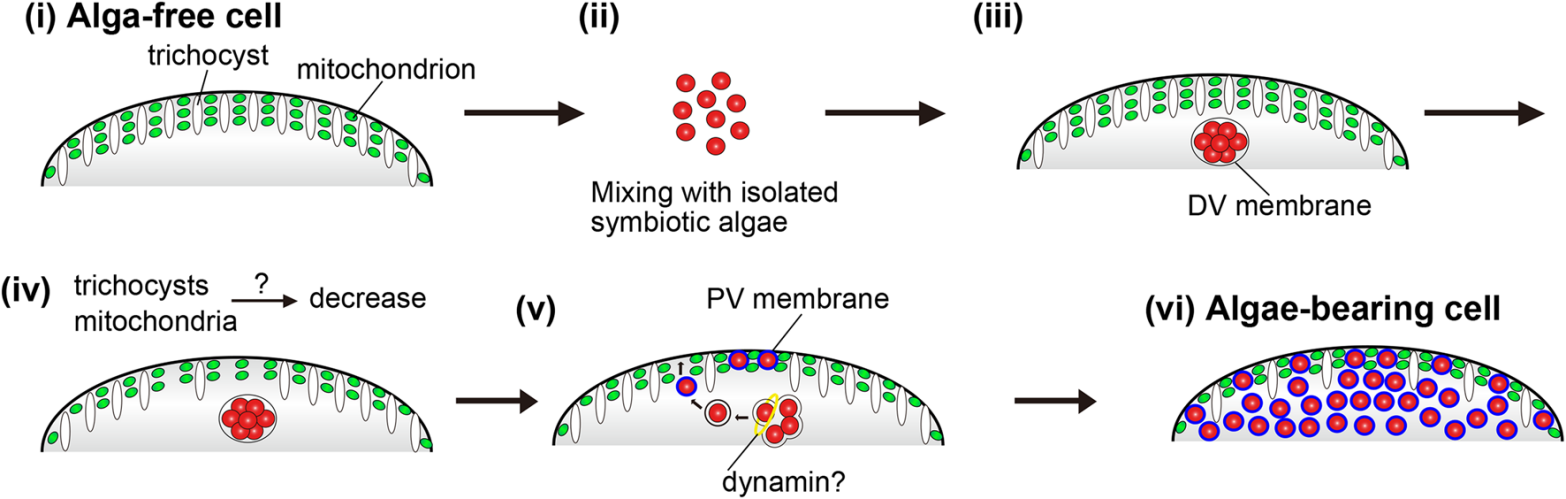
A schematic diagram of the algal attachment mechanism beneath the host cell cortex during the early alga reinfection. Before mixing with the symbiotic algae, many mitochondria and trichocysts were observed under the cell cortex of alga-free *P. bursaria* (i). After mixing with algae (ii), the algae are wrapped with the DV membrane (iii). (iv) Then, the trichocysts under the cell cortex are digested, as shown in our previous study^22^. Mitochondrial number may be decreased at the same timing. (v) The alga enclosed by the PV membrane moves toward the host cell cortex for attachment to the trichocyst and mitochondria-less area after the DV-budding related to dynamin^20^. (vi) Consequently, algal reinfection process was completed.

Figure 6 shows the schematic representation of the algal attachment mechanism beneath the host cell cortex during the early algal reinfection process. This representation can be defined by our immunofluorescence microscopies using mAbs against both mitochondria and trichocysts. This is the first report that shows the change in the distribution of both mitochondria and trichocysts associated with the endosymbiotic algal localization beneath the host cell cortex. This can be explained in six points. (i) Before mixing with the symbiotic algae, *P. bursaria* has many mitochondria and trichocysts under the cell cortex. (ii) After mixing with the algae, (iii) the algae are ingested and are wrapped with the DV membrane. Both mitochondria and trichocysts still show no remarkable changes. (iv) Three hours after mixing with the algae, trichocysts under the *Paramecium* cell cortex are digested as a result of the host lysosomal fusion as showed in our previous study^22^. Mitochondrial number may be decreased at the same timing, although the decreasing mechanism is still unknown. (v) After the DV-budding related to dynamin^20^, the alga enclosed by the PV membrane moves toward the host cell cortex for attachment to the trichocyst-less area^22^. (vi) Consequently, algal reinfection was completed and an algae-bearing cell is established.

All oxygenic photosynthetic organisms, including algae, have an evolved P700 oxidation system. In response to high light and low CO_2_ conditions, the primary electron donor in photosystem I (PSI), P700, is oxidized to suppress the production of ROS, which could oxidatively inactivate the function of PSI^52,53^. The symbiotic *Chlorella* spp. in *P. bursaria* might also retain the P700 oxidation system, although this has not yet been established. Thus, it is possible that symbiotic *Chlorella* spp. supply the inactivation of ROS because the algae also have an evolved P700 oxidation system; however, our study provides the first evidence that by the decrease of number of host mitochondria in the presence of a symbiont may cause the reduction of the amount of ROS in algae-bearing cells.

Algae-bearing *P. bursaria* are significantly larger than alga-free cells^54^. Interestingly, the quantification of total protein extract of host *P. bursaria* with or without symbiotic algae showed that the protein concentration of alga-free *P. bursaria* cells was significantly higher than that of algae-bearing cells (Fig. 5a). The reduced total protein contents of the algae-bearing *P. bursaria* cells may be related to the reduction of the numbers of membraned organelle of algae-bearing cells such as mitochondria as shown in Fig. 5b and trichocysts as shown in our previous study^22^. Furthermore, we have found that digestive activity of algae-bearing *P. bursaria* cells is highly suppressed than that of the alga-free cells, and the algae-bearing cells cannot digest as many bacteria as the alga-free cells (Kodama and Fujishima, in preparation). Furthermore, previous transcriptome analysis between host *P. bursaria* with and without symbiotic *C. variabilis* showed the downregulation of ribosomal protein expression in symbiont-bearing *P. bursaria* cells, suggesting that algal proteins with functions equivalent to those of the host *Paramecium* cells are transferred to the host through the PV membrane, which consequently reduces the transcriptional activity of the host^34^. Our result of the quantification of total protein extract supports the transcriptome analysis.

A phenomenon that might explain the reduction in the number of mitochondria in the host has been observed. In *Paramecium*, a lot of mitochondria are found around organelles that require considerable energy utilization, such as basal bodies of the cilia, food granules, DVs, and contractile vacuoles, as well as near the cell cortex^55^. Alga-free *P. bursaria* swim longer distances compared to algae-bearing cells. The swimming distance and velocity of alga-free and algae-bearing *P. bursaria* need to be analyzed in future studies*.*

The following question arose from the present study. How does the number of mitochondria in *Paramecium* decrease during algal endosymbiosis? Bisharyan and Clark reported that the ciliates *Tetrahymena thermophila* and *Ichthyophthirius multifillis* can discard intact mitochondria to the extracellular space by triggering clusters of GPI-linked surface proteins or heat shock^56^. Their results suggest that the number of mitochondria in these ciliates can easily change.

Overall, our study provides credible evidence that the symbiosis of *C. variabilis* in *P. bursaria* induces a reduction in the number of host mitochondria. In the process which engulfed alga is acquired as plastids, there are four stages that Inouye and Okamoto have summarized in detail^57^. In stage I, the alga engulfed by the host cell is repeatedly taken up by the host cell as a temporal symbiont. In stage II, the symbiont and host cell divide synchronously, and the nuclei and mitochondria of the symbionts are retained. In stage III, mitochondria of the symbiont are erased one by one, but a vestigial symbiont nucleus is retained as a nucleomorph. In stage IV, the symbiont nucleus is erased, and only algal plastids are retained. Here, since mitochondrial erase as have shown in stage III also is occurring in algae-bearing *P. bursaria* cells, it is possible that the organellization of symbiotic algae may be progressing in *P. bursaria*. Although the endosymbiosis between *P. bursaria* and *Chlorella* spp. is not obligatory, the presence of symbiotic algae affects the number of host organelles. Since the number of mitochondria is higher in alga-free *P. bursaria* cells, reducing the host mitochondria is reversible. The mechanism of reduction in the number of host mitochondria caused by algal endosymbiosis needs to be investigated in future studies, which may help elucidate the mechanism by which symbionts become organelles in the process.

## Data Availability

The datasets used during the current study are available from the corresponding author on reasonable request.
